# Anti-HPV16 E2 Protein T-Cell Responses and Viral Control in Women with Usual Vulvar Intraepithelial Neoplasia and Their Healthy Partners

**DOI:** 10.1371/journal.pone.0036651

**Published:** 2012-05-09

**Authors:** Simon Jacobelli, Fedoua Sanaa, Micheline Moyal-Barracco, Monique Pelisse, Sophie Berville, Pascale Villefroy, Marie Odile North, Suzanne Figueiredo, Bénédicte Charmeteau, Thierry Clerici, Françoise Plantier, Françoise Arnold, Antoine Touzé, Nicolas Dupin, Marie-Françoise Avril, Jean-Gérard Guillet, Rémi Cheynier, Isabelle Bourgault-Villada

**Affiliations:** 1 Institut Cochin, Université Paris Descartes, CNRS (UMR 8104), Paris, France; 2 INSERM, U1016, Paris, France; 3 AP-HP, Hôpital Cochin, Paris, France; 4 INSERM, U618, Tours, France; 5 AP-HP, Hôpital Ambroise Paré, Boulogne Billancourt, France; 6 UVSQ, Versailles, France; The University of Hong Kong, Hong Kong

## Abstract

T-cell responses (proliferation, intracellular cytokine synthesis and IFNγ ELISPOT) against human papillomavirus 16 (HPV16) E2 peptides were tested during 18 months in a longitudinal study in eight women presenting with HPV16-related usual vulvar intraepithelial neoplasia (VIN) and their healthy male partners. In six women, anti-E2 proliferative responses and cytokine production (single IFNγ and/or dual IFNγ/IL2 and/or single IL2) by CD4+ T lymphocytes became detectable after treating and healing of the usual VIN. In the women presenting with persistent lesions despite therapy, no proliferation was observed. Anti-E2 proliferative responses were also observed with dual IFNγ/IL2 production by CD4+ T-cells in six male partners who did not exhibit any genital HPV-related diseases. *Ex vivo* IFNγ ELISPOT showed numerous effector T-cells producing IFNγ after stimulation by a dominant E2 peptide in all men and women. Since the E2 protein is absent from the viral particles but is required for viral DNA replication, these results suggest a recent infection with replicative HPV16 in male partners. The presence of polyfunctional anti-E2 T-cell responses in the blood of asymptomatic men unambiguously establishes HPV infection even without detectable lesions. These results, despite the small size of the studied group, provide an argument in favor of prophylactic HPV vaccination of young men in order to prevent HPV16 infection and viral transmission from men to women.

## Introduction

Human papillomaviruses 16 (HPV16) is involved in more than 50% of uterine cervical cancers and is the second cause of mortality by cancer in women worldwide, with an incidence of 500 000 cases and 230 000 deaths annually [1], [2]. These tumors are preceded by grade 3 intraepithelial neoplasia (CIN3), diagnosed on the basis of Pap smears and followed up by colposcopy and biopsies. We were interested by usual vulvar intraepithelial neoplasia (VIN) lesions which are HPV-related and commonly resemble persistent anogenital warts that are often multifocal pigmented papular lesions. Usual VIN is characterized by the presence of poorly differentiated or undifferentiated basal cells and highly atypical squamous epithelial cells [3]. The involvement of the entire thickness of the epithelium defines its grade 3 (VIN3), in which high-risk HPV (HR-HPV) types, essentially HPV16, play a direct role in up to 91% of the cases [4]. The viral E2 protein is a major regulator of viral replicative cycle. It is required, together with the E1 helicase, for both regulation of transcription and replication of viral DNA [5]. In contrast, E2 protein is generally undetectable in cancer due to preferential integration of the viral genome in the cell genome and disruption of the E2 open reading frame [6], [7]. Therefore E2 protein is a marker of viral infection specific of the early stages of viral gene expression in infected cells. This has formally been demonstrated in a recent work that showed a strong staining of the E2 protein in the intermediate differentiated layers of HPV16-infected tissues and low grade CIN of the cervix [8]. These data indicate that HPV16 E2 is highly expressed in low grade lesions and therefore represents a marker for HPV infection even before any clinical manifestation. The immune response specific of the two oncogenic viral proteins E6 and E7 has already been analyzed in studies aiming at understanding the local immune microenvironment in VIN as well as in cervical cancer and their precursor stages [9]–[11]. However these viral proteins, although probably expressed from the early stages of replicative cycle, may be expressed at levels too low to be useful markers for HPV infection. Previous experiments done with HPV16 E6 and E7 in VIN indicated that the blood-T cell responses against these proteins are very low [12]–[15] and we predicted that the response toward an earlier more abundant viral protein would be higher. Previous experiments performed in women infected by HPV16 with or without HPV16-induced vulvar or cervical lesions strengthen this hypothesis although no time course study was performed and results were sometimes controversial. Blood proliferative T-cell responses against HPV16 E2 peptides were observed in 50% of healthy women, who presumably cleared HPV16 infection previously [16] and in 9 out of 22 regressive CIN3 cases [17]. In another study, 50% of women affected with usual VIN showed anti-E2 proliferative responses [18]. In contrast, the lack of anti-E2 proliferative responses was reported in 16 out of 18 patients (89%) affected with usual VIN lesions [19] and in 7 out of 8 and in 9 out of 12 women affected with CIN3 [16], [17]. The aim of the present study was to longitudinally follow, over an 18 months period, anti-E2 blood T cell responses in 8 women presenting with usual HPV16-related VIN in order to search for a relationship between blood anti-E2 T-cell responses and healing. We also analyzed HPV infection and anti-HPV16 E2 blood T-cell responses in their asymptomatic male partners who were regularly and chronically exposed to HPV16 during sexual intercourses with their wives. Indeed, it has been previously demonstrated that men are vectors of oncogenic HPV infection [20]. However, while HPV infection was found in 71 to 90% of the partners of women presenting with HPV infections [21], [22], only 52% harbored the same HPV subtypes [23]. Moreover, penile intra-epithelial neoplasia is rare and can only be detected in less than 2% of the men in contact with oncogenic HPV [24]. In the present study, we hypothesized that male partners exposed to replicative HPV16 could develop immunologic responses against the early E2 viral protein allowing control of infection.

## Results

### HPV16-E2 Specific Proliferative T-cell Responses in Women Correlate with the Evolution of the Lesion

Eight women presenting with VIN were included in the study. Two were treated and cleared their lesions within a few months before study entry (F#2 and F#4), four were symptomatic at study entry and cleared their VIN during the study (F#3, F#5, F#6 and F#8) while two had persistent lesions despite therapy (F#1 and F#7).

**Figure 1:**
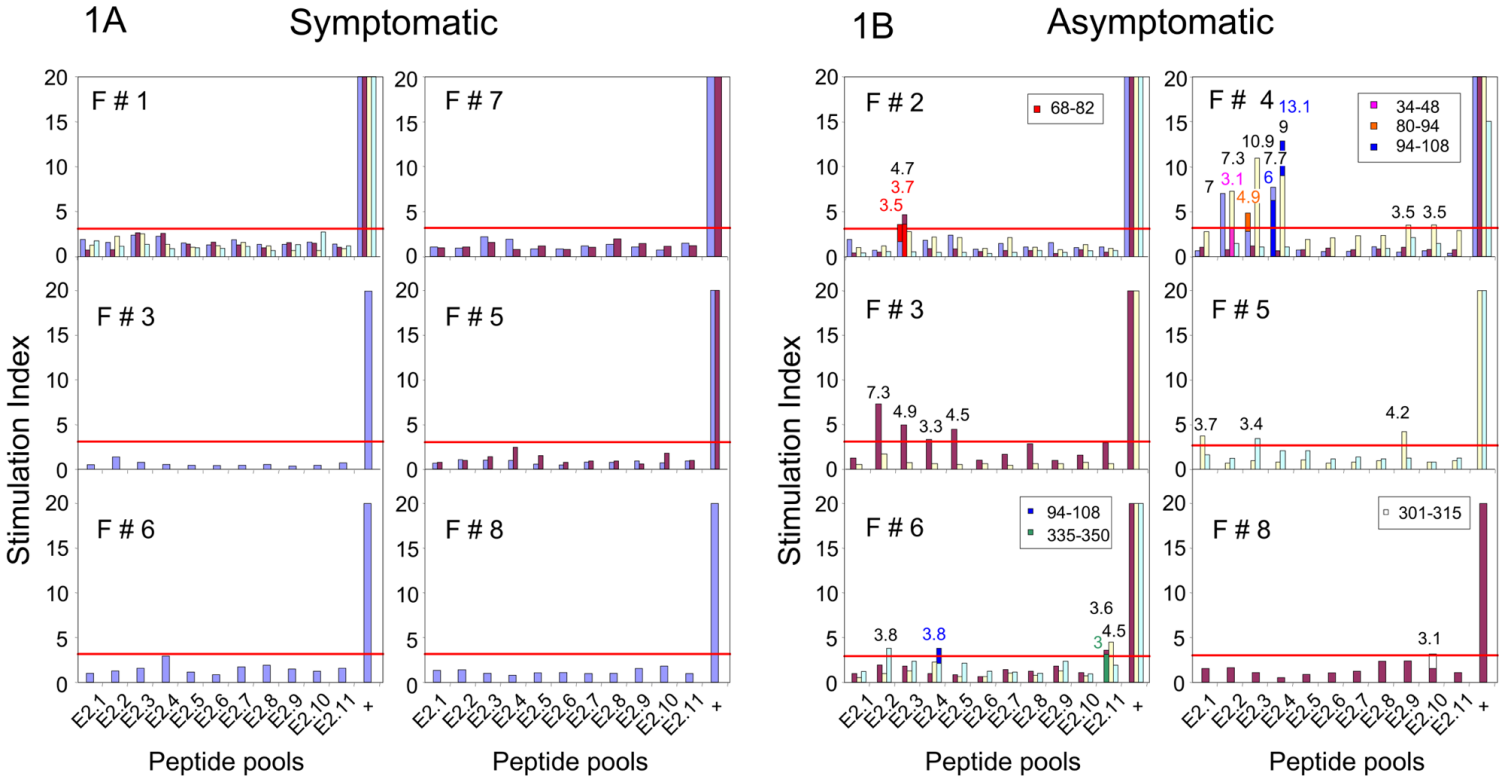
Anti-HPV16 E2 peptide proliferative responses in women. T-cell proliferation induced by E2 peptide pools were studied with PBMCs from A: women presenting with usual VIN either during the whole study (F#1 and F#7) or at study entry and up to the healing after treatment (F#3, F#6, F#8 before M6, F#5 before M12) and B: asymptomatic women having cleared their usual VIN after treatment (F#2, F#4 before their entry in the study, F#3, F#6, F#8 from M6, F#5 from M12). SI is represented by the cpm in peptide-stimulated cells/cpm in negative control wells (without peptide). Each bar represents SI against a pool of E2 peptide or positive control (+) obtained from PBMCs sampled at M0 (blue), M6 (red), M12 (yellow) and M18 (turquoise blue). Proliferative responses with SI >3 were scored as positive, provided that cpm in the negative control was above 500. The values of positive SI for E2 pools are indicated in black near the corresponding bar. Responses to 15 mer peptides comprised in the various peptide pools are shown, superposed to the response to the pool, in another color. The SI values for these peptides are indicated using the same colors near the corresponding bars.

In these two latter women (F#1, F#7) with persistent VIN lesions during the whole study, E2 peptide specific proliferative T-cell responses remained undetectable (Figure 1A).

In the four women (F#3, #5, #6, #8) who presented with usual VIN lesions at entry in the study and cleared them under treatment, no proliferative responses were observed at early stages (Figure 1A) whereas they became detectable at the time of clinical clearance of the lesions (at month 6 (M6) for F#3, #6, #8 and at M12 for F#5) (Figure 1B). These responses were directed against E2-1 to E2-5, E2-9 to E2-11 pools, with stimulation index (SI) ranging from 3 to 7.3. Peptides 94–108 (SI = 3.8) for E2–4 pool and 335–350 (SI = 3) for E2-11 pool were individualized as the recognized peptides in F#6 while peptide 301–315, included in E2-10, was recognized (SI = 3.1) by F#8. These responses were most probably mediated by CD4+ T-cell as they were observed in CD4+ T lymphocyte-enriched fractions in F#3 (data not shown).

Similarly, positive responses were observed in the two women who presented with VIN lesions three and four months before entering the study but were asymptomatic after therapy at entry (F#2, #4) (Figure 1B). The E2-3 pool was recognized in these two women with SI of 4.7 and 10.9 at M6 and M12 respectively. Two reactive 15-mer peptides were identified in this pool: peptide 68–82 and peptide 80–94 recognized with SI of 3.7 and 4.9, respectively. E2–2, E2–4, E2–9 and E2–10 pools were also targeted by T lymphocytes from F#4. Peptide 34–48 (SI = 3.1) was identified in the E2–2 pool and peptide 94–108 (SI = 13.1) in the E2–4 pool.

Patients who cleared VIN before or during follow up displayed a strong proliferative T-cell response (6/6), while such a response was undetectable in women having classic VIN lesions (0/6) (p = 0.002, Fisher exact test).

### HPV16-E2 Specific Proliferative T-cell Responses in Male Partners

**Figure 2:**
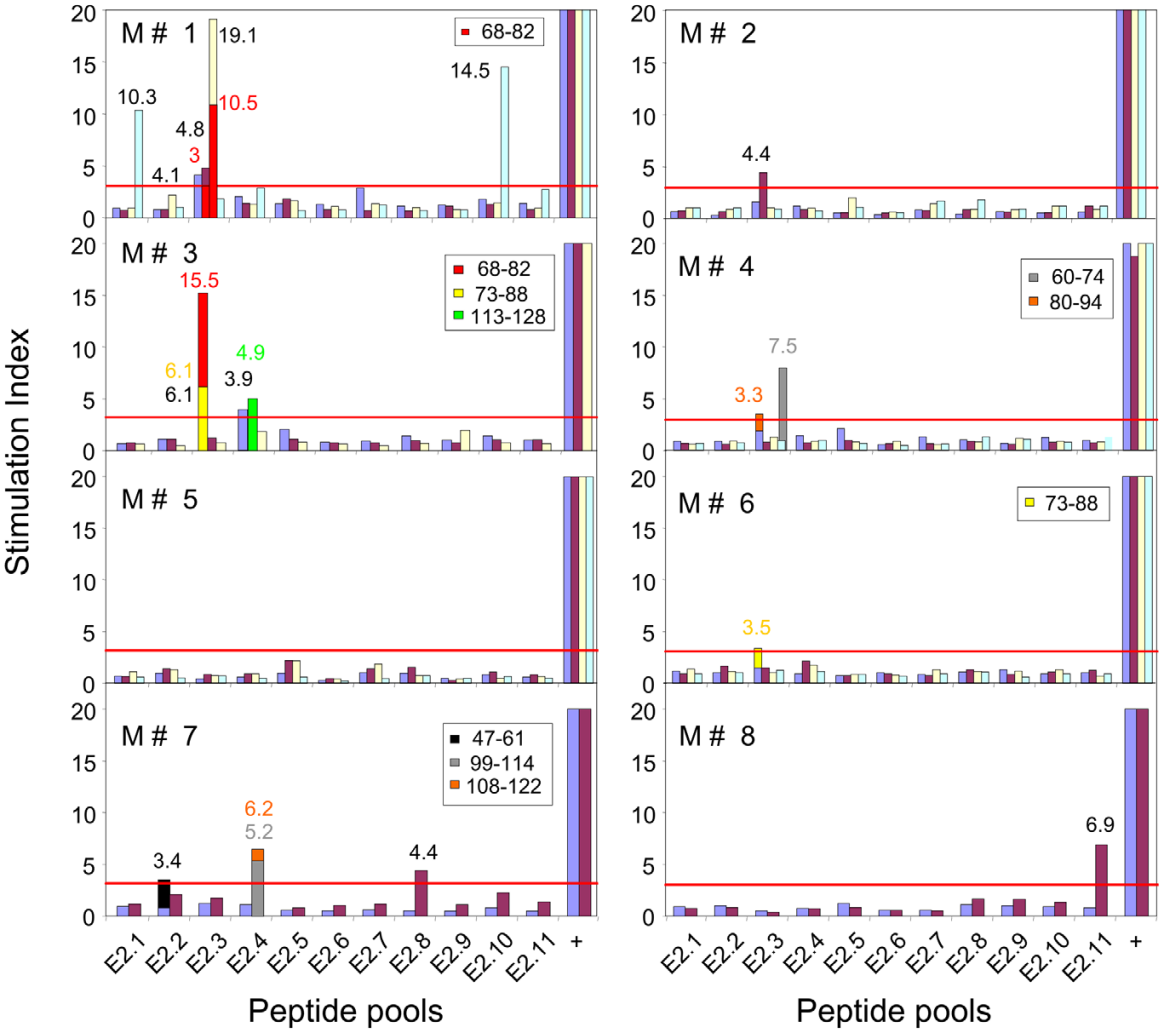
Anti-HPV16 E2 peptide proliferative responses in male partners of women presenting with usual VIN. Proliferative assays were performed using PBMCs in presence of pools of E2 peptides. SI is represented by the cpm in peptide-stimulated cells/cpm in negative control wells (without peptide). Each bar represents SI against a pool of E2 peptide or positive control (+) obtained from PBMCs sampled at M0 (blue), M6 (red), M12 (yellow) and M18 (turquoise blue). Proliferative responses with SI >3 were scored as positive, provided that cpm in the negative control was above 500. The values of positive SI for E2 pools are indicated in black near the corresponding bar. Responses to 15 mer peptides comprised in the various peptide pools are shown, superposed to the response to the pool, in another color. The SI values for these peptides are indicated in the same colors near the corresponding bars.

None of the eight partners presented with any HPV lesions and HPV16 was undetectable by genital brushing and HPV DNA typing in all but one (M#8) cases (Table 1). All but one (M#5) partners of the women with usual VIN lesions had proliferative responses against E2 peptides (Figure 2). Cell proliferations were observed after stimulation by peptides from E2-3 pool in five of them (M#1, #2, #3, #4, #6), by peptides from E2-4 in two (M#3, #7) and by peptides from E2-1 in M#1, E2-2 in M#7, E2-8 in M#7, E2-10 in M#1 and E2-11 in M#8. The most strongly stimulating peptide in the E2-3 pool was peptide 68–82 with SI = 10.5 and 15.5 in M#1 and M#3 respectively. Other peptides recognized in the E2-3 pool were peptide 73–88 (SI = 6.1 and 3.5 in M#3 and M#6 respectively), peptide 60–74 (SI = 7.5 in M#4) and peptide 80–94 (SI = 3.3 in M#4). In E2-4 and E2-2 pools, peptides 99–114 (SI = 5.2 in M#7), 108–122 (SI = 6.2 in M#7), 113–128 (SI = 4.9 in M#3) and 47–61 (SI = 3.4 in M#7) were also identified as immunogenic peptides.

**Table 1:** Clinical and biological characteristics of the eight women having usual VIN and their male partners.

Sex	Couple	Age(years)	Time elapsedfrom VIN onset(months)	HPVtyping	Presence ofgenital lesionsat time of inclusion	Presence of genital lesions between months 6 and 18	Previous VINtreatment	Previous cervicalHPV disease	OtherHPV disease	HLA	Titers ofanti-HPV antibodies
F	1	33	84	16	yes	yes	laser, imiquimodsurgical excision	no	common warts	A2/33 B41/58DR3/15	1∶40
F	2	54	180	16	no for 3 months	no	laser, imiquimod	no	no	A68/− B15/40DR4/15	1∶ 20
F	3	39	5	16	yes	no at 6 months	imiquimod	no	no	A11/24 B15/35DR13/16	1∶ 20
F	4	38	18	16	no for 4 months	no	Electrocoagulation	no	common warts	A2/3 B35/57DR4/11	1∶ 160
F	5	62	11	16	yes	no at 12 months	imiquimodcryotherapy	no	common warts	A24/29 B44/51DR7/15	negative
F	6	55	132	16	yes	no at 6 months	laser, imiquimod	invasive cervicalcarcinoma	common warts	A2/24 B13/35DR4/11	1∶ 40
F	7	37	28	16	yes	yes	laser, imiquimodtopical 5-FU	CIN3	common warts	A1/3 B35/57DR4/7	1∶ 80
F	8	50	9	16	yes	no at 6 months	imiquimod	no	no	A24/32 B40/44DR4/7	negative
M	1	36	NA	negative	no	no	NA	NA	common warts	A11/23 B45/45 or 50 DR4/10	1∶ 40
M	2	66	NA	negative	no	no	NA	NA	no	A11/23 B35/49DR11/11	negative
M	3	40	NA	negative	no	no	NA	NA	no	A30/33 B42/45DR3/8	1∶ 40
M	4	41	NA	negative	no	no	NA	NA	common wartslaryngeal papillomatosis	A2/68 B35/51DR3/−	negative
M	5	68	NA	27	no	no	NA	NA	no	A2/11 B18/40DR7/16	negative
M	6	58	NA	negative	no	no	NA	NA	no	A29/68 B35/44DR7/13	1∶ 20
M	7	35	NA	negative	no	no	NA	NA	no	A02/24 B63/51DR8/13	1∶ 20
M	8	56	NA	16	no	no	NA	NA	no	A02/32 B40/44DR1/4	1∶ 20

NA: not applicable.

In M#1 and M#4, proliferative responses were observed in CD4+ T lymphocyte-enriched fractions or in CD8-depeleted T-cell fraction (data not shown).

Taken together, these data indicate that, despite the absence of detectable HPV-related lesions, all male partners but one (M#5) showed E2-specific proliferative T cell responses. It is important to note that M#5 had rheumatoid arthritis treated by low doses of methotrexate (10 mg per week) and corticosteroids (8 mg per day) for 10 years.

### Intracellular Cytokine Staining in Proliferative T-cell Responder Women

**Figure 3:**
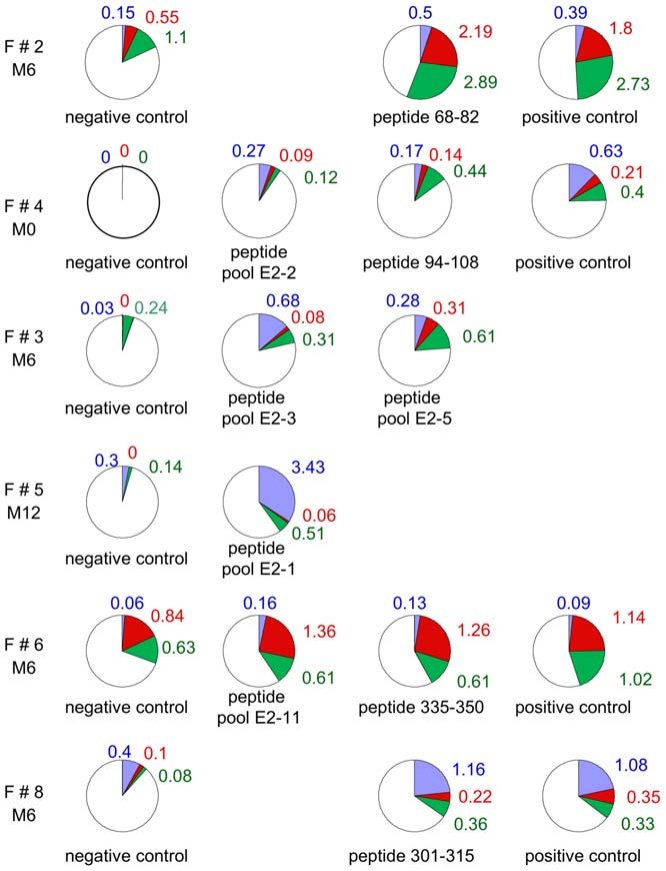
Intracellular cytokine synthesis to HPV16 E2 peptides in proliferative T-cell female responders. The percentage of CD4+ T-cells synthesizing single IFNγ, dual IFNγ/IL2 or single IL2 was measured in response to E2 peptide pools or E2 peptide and compared to negative (no or irrelevant peptide) and positive controls (pool of non-HPV viral peptides). Single IFNγ responses are shown in blue, dual IFNγ/IL2 in red and single IL2 in green. Percentages of CD4+ responding cells are in the same color as the cytokine(s) synthesized. The white color represents the percentage of non responding cells among CD4+ T lymphocytes. Responses were considered as positive when the percentage of CD4+ T-cells synthesizing cytokines increased by at least 0.2% in the presence of the peptide as compared to negative control.

In order to further characterize the E2-specific responses in patients clearing usual VIN after treatment, we analyzed IFNγ and IL2 production by HPV16 E2-stimulated CD4+ T-cells from women whose peripheral blood mononuclear cells (PBMC) displayed positive proliferative responses (F#2, #3, #4, #5, #6 and #8). In all six women, intracellular staining for IFNγ and/or IFNγ/IL2 and/or IL2 by CD4+ T lymphocytes was observed in the presence of at least one peptide or a pool of peptides identified by proliferation tests (Figure 3). IFNγ only was synthesized in the presence of peptide 68–82 by F#2, of pool E2-2 by F#4, of pool E2-3 or peptide E2-5 by F#3, of pool E2-1 by F#5 and of peptide 301–315 by F#8. Dual IFNγ/IL2 response after stimulation by one peptide or a pool of peptide was observed in women F#2 (peptide 68–82), who entered the study after tumor treatment and in women F#3 (pool E2-5), F#6 (pool E2-11 and peptide 335–350) and F#8 (peptide 301–315) at the time of the clearance of their lesion. Synthesis of IL2 alone was observed in F#2 (peptide 68–82), F#4 (peptide 94–108), F#3 (pool E2-5), F#5 (pool E2-1) and F#8 (peptide 301–315).

### Intracellular Cytokine Staining in T-cell Responder Male Partners

**Figure 4:**
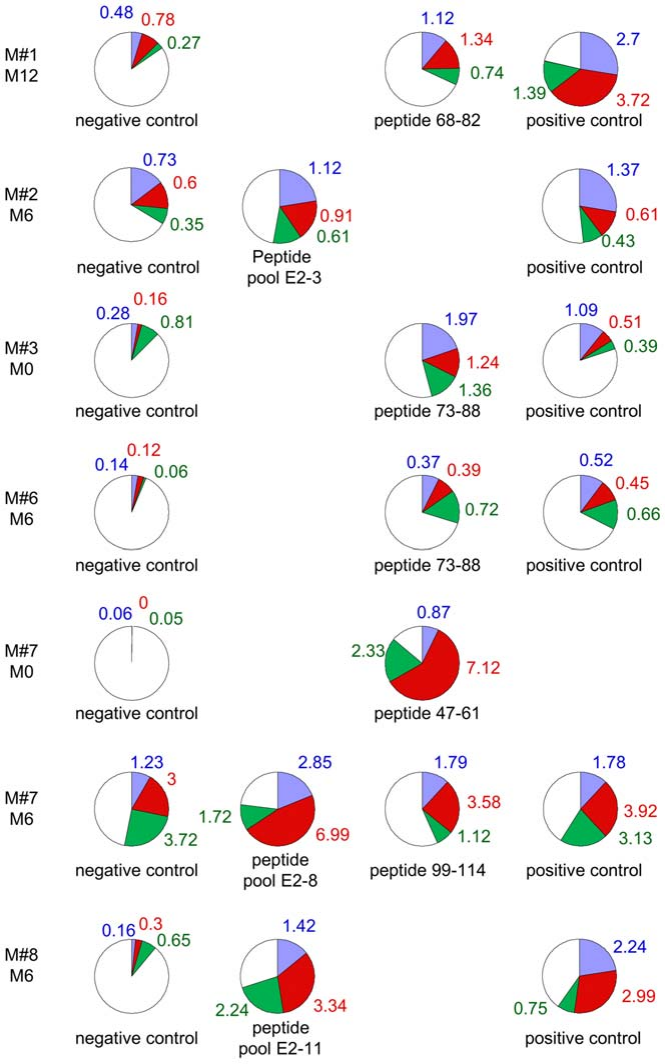
Intracellular cytokine synthesis to HPV16 E2 peptides in proliferative T-cell **male responders.** The percentage of CD4+ T-cells synthesizing single IFNγ, dual IFNγ/IL2 or single IL2 was measured in response to E2 peptide pools or E2 peptides and compared to negative (no or irrelevant peptide) and positive controls (pool of non-HPV viral peptides). Single IFNγ responses are shown in blue, dual IFNγ/IL2 in red and single IL2 in green. Percentages of CD4+ responding cells are shown using the same color as the cytokine(s) synthesized. The white color represents the percentage of non responding cells among CD4+ T lymphocytes. Responses were considered as positive if the percentage of CD4+ T-cells synthesizing cytokines was at least 0.2% higher in the presence of stimulating peptide(s) than in negative control.

Among the 7 male partners showing positive proliferative responses, synthesis of single IFNγ, dual IFNγ/IL2, and single IL2 by CD4+ T-cells in the presence of at least one peptide or a pool of peptides was evidenced in six cases (M#1, #2, #3, #6, #7, #8) (Figure 4). High percentages of such CD4+ T-cells were found in the partners in whom proliferative assays exhibited SI superior to 5 (peptide 68–82 in M#1, peptide 73–88 in M#3, peptide 99–114 in M#7, pool E2-11 in M#8). Intracellular cytokine production was undetectable in M#4.

### *Ex vivo* IFNγ ELISPOT assays in Usual VIN Women

**Figure 5:**
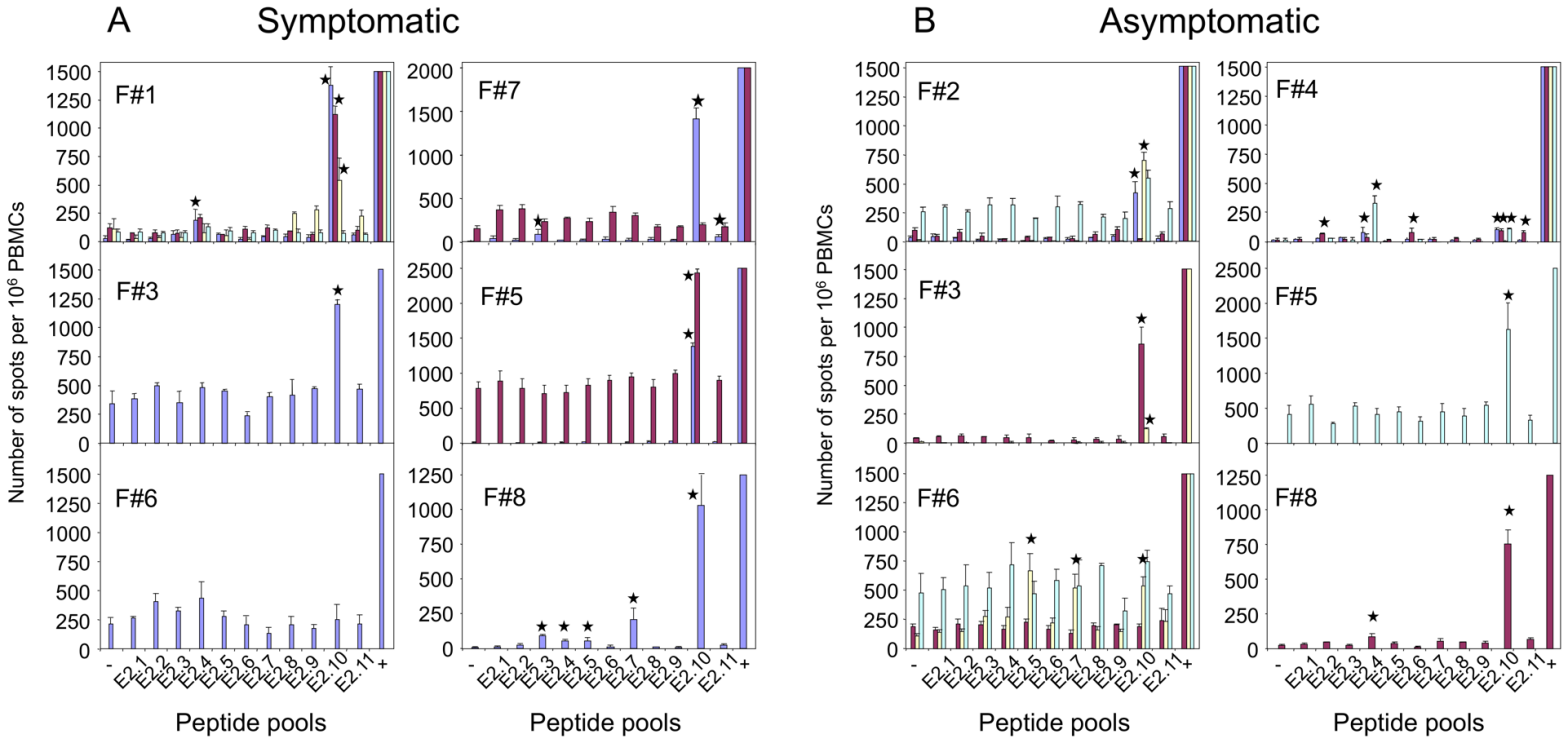
*Ex vivo* anti-HPV16-E2 peptide IFNγ ELISPOT responses in women presenting with usual VIN. IFNγ ELISPOT response to E2 peptide pools was studied using PBMCs from A: women presenting with usual VIN either during the whole study (F#1 and F#7) or at study entry and up to the healing after treatment (F#3, F#6, F#8 before M6, F#5 before M12) and B: asymptomatic women having cleared their usual VIN after treatment (F#2, F#4 before their entry in the study, F#3, F#6, F#8 from M6, F#5 from M12). Bars represent numbers of SFC/10^6^ PBMCs against a pool of E2 peptides, negative (0) and positive (+) controls obtained at M0 (blue), M6 (red), M12 (yellow) and M18 (turquoise blue). Standard deviation of triplicates appears for each bar. Responses were considered significant when the mean number of SFC per 10^6^ cells in the 3 experimental wells was >3-fold the mean number of SFC in the negative control wells (PBMC alone) and >30 SFC/10^6^ cells. Positive responses are identified by a black star above the corresponding bar.

IFNγ ELISPOT responses were found in symptomatic women (Figure 5A), including F#1 and F#7 who suffered from persistent VIN lesions, and in three women before the time of clinical clearance under treatment (F#3, F#5, F#8). More than 1000 SFC/10^6^ PBMC against E2-10 were observed in these symptomatic women at different time-points, reflecting a high number of specific effector IFNγ-producing T cells. Significant IFNγ ELISPOT response contemporary to clinical VIN lesions was not detected in F#6 only. IFNγ ELISPOT were also found in all asymptomatic women (Figure 5B), including F#2 and F#4 who were asymptomatic at study entry and in four women after clinical clearance (F#3, F#5, F#6, F#8), with a number of cells below 850/10^6^ PBMC in all but one patients (F#5).

Three women had IFNγ ELISPOT responses against one pool of peptide (F#2, #3 and #5), one against two pools (F#1), two against three pools (F#6 and #7) and two against five pools (F#4 and #8) (Figure 5A and 5B). Among these pools, E2-10 was recognized by cells from all women, with more than 1000 spot forming cells (SFC)/10^6^ PBMC in five women (F#1, #3, #5, #7, #8), 500 to 1000 SFC in two women (F#2, #6) and about 100 SFC in the last case (F#4). In this E2-10 pool, peptide E2 309–323 was always the recognized peptide (data not shown).

### *Ex vivo* IFNγ ELISPOT assays in Male Partners

**Figure 6:**
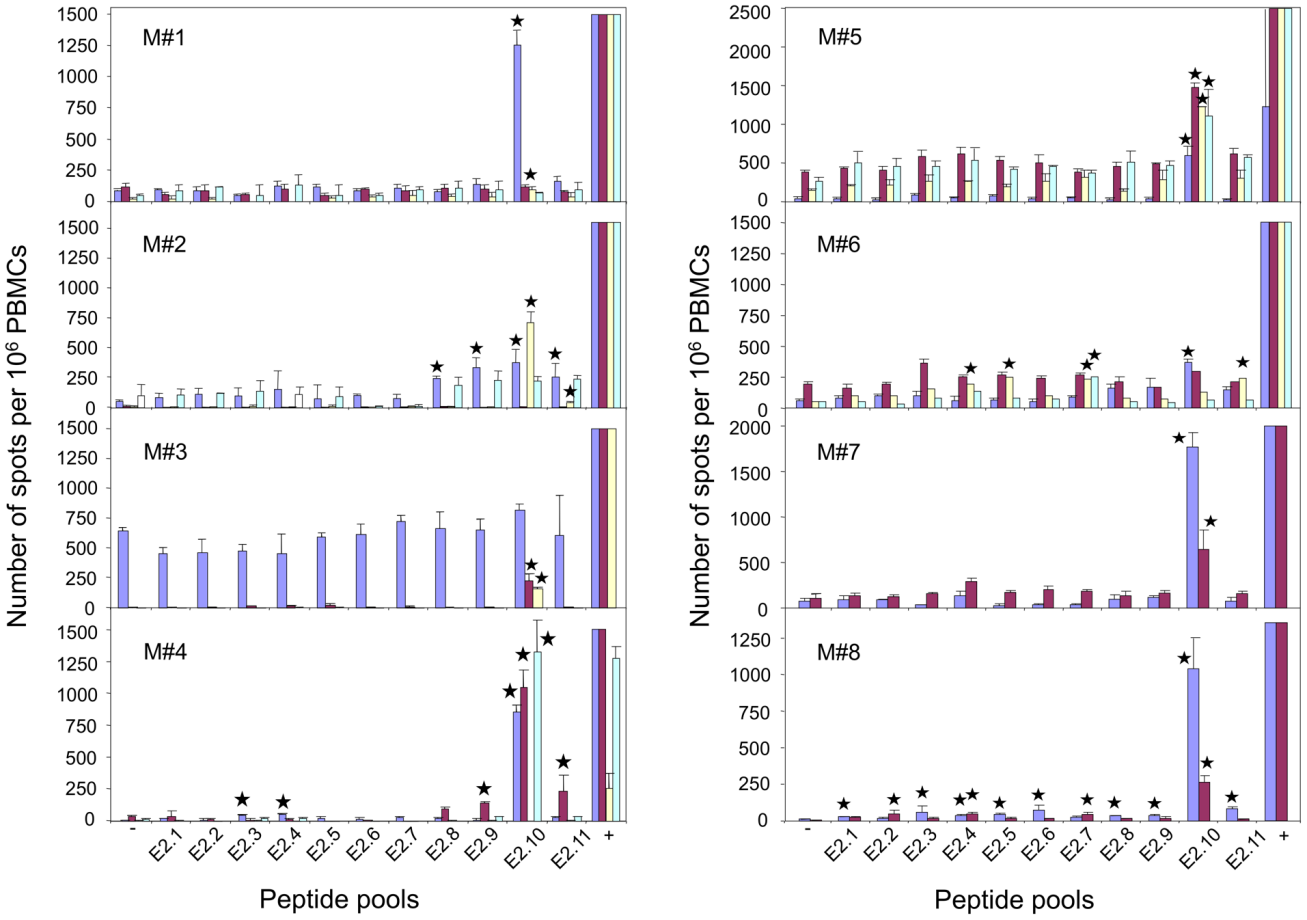
*Ex vivo* anti-HPV16-E2 peptide IFNγ ELISPOT responses in male partners of women presenting with usual VIN. IFNγ ELISPOT responses to E2 peptide pools were studied with PBMCs from male partners of women presenting with usual VIN. Bars represent numbers of SFC/10^6^ PBMCs against a pool of E2 peptides, negative (0) and positive (+) controls obtained at M0 (blue), M6 (red), M12 (yellow) and M18 (turquoise blue). Standard deviation of triplicates appears for each bar. Responses were considered significant when the mean number of SFC per 10^6^ cells in the 3 experimental wells was >3-fold the mean number of SFC in the negative control wells (PBMC alone) and >30 SFC/10^6^ cells. Positive responses are identified by a black star above the corresponding bar.

PBMCs from all the male partners exhibited a positive response after stimulation by one pool in four of them (M#1, #3, #5, #7), four pools in one (M#2), five pools in two (M#4 and #6) and all eleven pools of E2 peptides in the latter case (M#8) (Figure 6). The immunodominant E2 peptides pool was E2-10 with a response greater than 1000 SFC/10^6^ PBMC in five individuals (M#1, #4, #5, #7, #8) and up to 700 SFC in another partner (M#2). PBMCs from M#3 and M#6 also showed positive responses against the same pool E2-10 with 230 and 400 SFC respectively. Peptide 309–323 was identified as the dominant peptide recognized in this E2-10 pool (data not shown). We therefore detected a strong anti-E2 response in all male partners, more specifically against a peptide located in the C-terminal part of the protein that was also immunodominant in the samples from the women partners.

## Discussion

In the present work, we studied HPV16 E2 protein because of its early and strong expression [8]. E2 is a larger protein than E6 and E7 proteins and it induces more T-cell responses than E6 and E7 [16], [25]. Therefore looking for an E2-specific response appeared to be more sensitive. In addition, E2 is required for viral replication [26] and the detection of E2-specific T cell responses is the signature of this event [25]. The study of anti-E2 T-cell responses is thus appropriate in the early phases of HPV16 infection or for pre-malignant lesions as usual VIN. We observed proliferative responses to HPV16-E2 antigens in 6 out of 8 women presenting with usual HPV16-associated VIN. Two of them were asymptomatic at study entry (treated 3 and 4 months before by laser or electrocoagulation, respectively) whereas in the four other women proliferative responses strongly linked with ongoing clearance of the lesion after treatment (imiquimod or laser). In contrast, no E2-specific responses were detected in patients with persistent lesions and in the four other ones before they underwent VIN treatment. Then the association between the disappearance of the HPV-16 induced vulvar lesions under therapy and the development of E2-specific proliferative T cell responses is striking. The appearance of anti-E2 proliferative T-cell responses could be a consequence of the release of E2 protein after local therapy, as they did not occur spontaneously.

The six women who demonstrated proliferative responses also had CD4+ T-cells synthesizing IL2 alone, IFNγ alone or both IFNγ and IL2. Such a profile (presence of proliferating and cytokine-producing polyfunctional population) suggests that these CD4+ T-cell responses are anti-E2 memory CD4+ T-cell responses as previously described by Harari [27].

Moreover, all women, whether they cleared or not their lesion after treatment, had very high number of anti-HPV16-E2 T-cells as tested by *ex vivo* IFNγ ELISPOT. The presence of a monofunctional population producing IFNγ only, without detectable proliferation observed in the women presenting with persistent lesions (F#1, #7 and #3, #5, #6, #8 before treatment) is consistent with the presence of effector T-cells and an uncontrolled HPV16 infection. This is in agreement with previous study showing that the detection of specific immune responses against E2 peptides by IFNγ ELISPOT did not correlate with either HPV16 control or VIN or CIN1 clearance [25]. Moreover, after local therapy and healing, the number of IFNγ-producing T-cells decreased, as observed in women F#3 and F#8. The latter observation suggests that the monofunctional T-cell population producing IFNγ only depends on the quantity of HPV16-E2 protein synthesized.

Strong anti-E2 IFNγ ELISPOT responses against HPV16 309–323 E2 peptide were found in all women. This 15-mer peptide, included in the E2-10 peptides pool, shares more than 70% amino acids (by BLAST analysis) only with cutaneous HPV2 and 27 and with mucosal HPV55. Five of eight women had a past history of common warts (F#1, #4, #5, #6, #7) (Table 1). In order to exclude a possible cross-reactivity between HPV types, we performed *ex vivo* IFNγ ELISPOT assays against E2 309–323 peptide from HPV2, 27 and 55 that were all negative (Table 2). Consequently, we conclude that the IFNγ ELISPOT responses against HPV16 309–323 E2 peptide are indeed specific for HPV16.

**Table 2:** Ex vivo IFNγ ELISPOT responses in women and men against HPV16, HPV2, HPV27 and HPV55 E2 309–323 peptide.

Number of SFC for 10^6^ PBMCs ± standard deviation^A^					
Patient	PoolE2-10HPV 16	Peptide309–323HPV 2	Peptide309–323HPV 16	Peptide309–323HPV 27	Peptide309–323HPV 55
F # 3	809±150	–^B^	845±78	–	–
F # 4	85±18	–	250±52	–	–
F # 5	1692±48	–	1692±52	–	–
F # 7	1399±63	–	1613±36	–	–
F # 8	1021±452	–	990±129	–	–
M # 3	160±18	–	114±54	–	–
M # 4	1307±243	–	1487±18	–	–
M # 5	1117±63	–	1117±31	–	–
M # 6	334±28	–	663±142	–	–
M # 7	1697±53	–	1463±43	–	–
M # 8	1033±306	–	266±109	–	–

A: number of SFC in negative control was substracted.

B: number of SFC was <3-fold the mean number of SFC in the negative control.

In the absence of condom usage for at least 6 months, the male partners of the eight women could be contaminated by HPV16. HPV16 and HPV27 (a cutaneous HPV) were identified in genital sampling gathered by cytobrushing in only two of the eight healthy partners (M#8, #5) (Table 1). Such a prevalence of HPV16 contamination is similar to the one usually observed in male partners of oncogenic HPV-infected women [23]. This does not exclude the possibility that the other male partners could also be infected either at undetectable levels or in anatomical sites not sampled by the cytobrush.

We also observed HPV16-E2-specific proliferative responses in seven male partners and intracellular synthesis of single IFNγ, dual IFNγ/IL2 and single IL2 in six of them. These E2 specific T-cell responses in all male partners but two (M#4, #5) indicate a striking link between the absence of HPV-related lesions and the presence of spontaneous E2-specific proliferative T-cell responses and single IFNγ, dual IFNγ/IL2, single IL2 T-cell production, as previously described in other viral systems [27], [28]. Such a spontaneous polyfunctional anti-E2 T-cell response could be due to an efficient presentation of viral antigens by dendritic cells present in mucosal tissue and it is tempting to speculate that E2-specific responses prevent HPV16-related lesions. Therefore, spontaneous HPV16 control could be related to the presence of polyfunctional memory CD4+ T-cells in male partners.

Peptide 309–323 was also the dominant peptide detected in IFNγ ELISPOT assay in male partners. Indeed, high frequencies (>500 SFC/10^6^ PBMC) of blood effector T-cells, directed against E2 309–323 peptide and synthesizing IFNγ alone without proliferative capacity were identified in the majority of male partners (6/8) by *ex vivo* anti-E2 peptide IFNγ ELISPOT assays. The level of this response with very high number of spots is similar to that observed during the chronic phase of HIV infection characterized by highly replicative viral reactivation [28], [29]. In the six men tested, there was no detectable cross reactivity with 309–323 E2 peptide from HPV2, 22 and 55, similarly to the findings in women (Table 2). Since E2 protein is not encapsidated, stimulation of anti-E2 cellular immune responses is a mark of the presence of viral replication in infected cells. The high frequency of E2-specific T-cells measured in partners of women with usual VIN demonstrates that the virus effectively replicates in males.

In summary, the analysis of E2-specific T-cell responses is a sensitive and reliable tool to analyze disease progression and the natural history of HPV infection and premalignant lesions. In women, polyfunctional T-cell responses featured by proliferative responses and cytokine synthesis reflect memory CD4+ T-cell population induced by VIN treatment. Monofunctional T-cell responses restricted to *ex vivo* IFNγ ELISPOT reflect the presence of numerous HPV16 E2-specific effector T-cells but does not correlate with control of HPV16 infection.

In the male partners, the presence of polyfunctional spontaneous T-cell proliferative responses with single IL2, dual IFNγ/IL2, and single IFNγ memory T-cells against HPV16 E2 peptides in the absence of detectable lesion despite HPV16 exposure suggests an efficient and spontaneous control of genital HPV lesions. These results are reminiscent of those described in Gambian prostitutes who, despite frequent exposure to HIV showed strong anti-HIV cytotoxic T-cell responses and remained uninfected [30]. Such anti-HPV16 T cells responses may thus reflect the control of viral infection.

We therefore demonstrate that, although not clinically detectable, HPV16 can replicate in men and induce a strong memory T cell response against one of the early viral proteins. Despite the fact that they were obtained in a small cohort of couples, our results suggest that males are an important reservoir of genital HPVs and provide an argument in favor of prophylactic HPV vaccination of young men with virus like particles in order to prevent HPV16 infection in men and thus fight against the spread of mucosal HPV diseases in the population.

## Materials and Methods

### Ethics Statement

Promotion of the trial was made by INSERM (RBM 05–48). Approbation of ethic committee from Ambroise Paré hospital was obtained December 6^th^, 2005 (# 05 11 74). Every male and female signed a written informed consent before starting the study.

### Inclusion and Exclusion Criteria of Couples

Every consecutive woman eligible for the study presented with usual multifocal VIN proved by biopsy and documented HPV16 infection. Their current regular male sex partners were also included. Additional inclusion criteria were sexual intercourses not protected by condoms for at least 6 months. Exclusion criteria were pregnancy, HIV seropositivity, CD4 T-cell count below 300/µl and hemoglobin below 10 g/dl.

### Female Patients

Eight 18–65 year-old (mean 46+11 years) consecutive women entered the study (Table 1). They were followed up by doctors M.MB, M.P and S.B at Cochin or Ambroise Paré Hospitals at the inclusion in the study (M0) and then every 6 months for 18 months (M6, M12, and M18). Their VIN symptoms had appeared 5 to 180 months (mean 58+66 months) prior to inclusion. They experienced numerous relapses despite multiple destructive treatments (cryotherapy, electrocoagulation, surgery or laser) or local topical therapy (5-fluorouracil, imiquimod). At study entry, two women (F#2, #4) had no detectable vulvar lesions since 3 and 4 months respectively, after a curative treatment by imiquimod and electrocoagulation. They did not show any recurrence during the follow-up period of 18 months. Two women (F#3, #8) started imiquimod therapy at study entry and cleared their usual VIN lesions during the next 6 months. Two patients (F#5, #6) received laser treatment either at the first visit (F#6) or one month before the third visit (F#5) and cleared their infection at M6 and M12 respectively. The two last patients (F#1, #7) had persistent lesions during the whole study. Patient F#7 was previously affected with CIN3 treated by surgery. Patient F#6 had invasive cervical carcinoma also treated by surgery. Five women had previously presented common warts (F#1, #4, #5, #6, #7). DR4 was the most frequent HLA class II antigen (5/8 women), followed by DR7 (3/8), DR15 (3/8) and DR11 (2/8). HPV-16 serological assay was negative in two women (F#5, F#8) and positive in the six other ones.

### Male Partners

No male partners (mean age 50+14 years) had HPV-related genital lesions at inclusion in the study and during follow-up (Table 1). Samples for HPV typing were obtained by genital brushing in distal urethra and prepuce internal surface, two areas where HPV is most frequently found [21]. HPV16 and HPV27 were found in M#8 and #5 respectively. Two men had previous history of HPV-related disease (common warts in M#1 and #4, laryngeal papillomatosis in M#4). One man (M#5) had rheumatoid arthritis treated by low doses of methotrexate (10 mg per week) and corticosteroids (8 mg per day) for 10 years. No predominant HLA class II allele was observed. HPV16 serological assay was negative in three males (M#2, M#4, M#5) and positive in the five others.

### Blood Samples

In accordance with the Ethics Committee of Ambroise Paré hospital, 150 ml blood samples were collected at study entry for each woman and her partner after informed consent. In most cases, blood samples were further collected at M6, M12, and M18. PBMC were isolated by centrifugation through lymphocyte separation medium (GE Healthcare, Amersham) and either immediately used or frozen in fetal calf serum (FCS)-10% DMSO and stored at –180°C in liquid nitrogen.

### HPV Typing

DNA extraction from keratinocytes obtained by genital brushing (men) or biopsy (women) was performed using the High Pure PCR Template Kit® (Roche). HPV infection was determined by PCR using the Universal L1 gene primers My09 and My11 [31]. Specific infection by HPV16 and HPV18 was assessed by specific primers. Positive and negative controls were DNA from an infected patient and water, respectively. PCR products were analyzed on 1.5% agarose gels, purified with ExoSapIt purification kit® (Amersham) and sequenced using the BigDye® Terminator v 1.1 cycle sequencing Kit (Appelera) on Sequencer Applied Biosystem 3130 XL ABI Prism 16 capillaries. The sequences were identified using the Basic Local Alignment Search Tool (BLAST) Nucleotid Algorithm.

### HPV16 Serological Assay

Flat-bottomed 96-well microplates (Nunc, Maxisorp) were coated with 200 ng per well of native virus like particles produced as previously described [32] in PBS1X-FCS10% at 37°C for 1 h. Sera were serially diluted from 1∶ 20 to 1∶ 2560 in PBS5X-FCS10%-2% Tween 20. After four washes with PBS1X-0.1% Tween 20, peroxidase-conjugated anti-human IgG Ab (Southern Biotech) diluted 1 : 20,000 was used in PBS5X-FCS10%-2% Tween 20 to detect the binding of human IgG. After four washes with PBS1X-0.1% Tween 20, revelation was done by ortho-phenylenediamine and H2O2. Colored reaction was stopped with 4N H2SO4 after 30 min at room temperature. Optic density (OD) was read at 490 nm. Every condition was tested in duplicate. Endpoint antibody titers were determined as the last of serial 2-fold dilution that yielded a positive result. The cut-off level above which absorbances were considered positive was 0.20.

### Synthetic Peptides

Fifty-three overlapping peptides (15 to 16-mer) (NeoMPS) spanning the whole E2 protein (365 amino acids) were used. Peptides were pooled as described in Table 3 (E2-1 to E2-11 pools).

**Table 3:** Peptides and pools spanning the whole E2 protein.

		1	2	3	4	5	6	7	8	9	10	11	12	13	14	15	16
E2-1	1–15	M	E	T	L	C	Q	R	L	N	V	C	Q	D	K	I	
	5–20	C	Q	R	L	N	V	C	Q	D	K	I	L	T	H	Y	E
	13–28	D	K	I	L	T	H	Y	E	N	D	S	T	D	L	R	D
	24–39	T	D	L	R	D	H	I	D	Y	W	K	H	M	R	L	E
	30–44	I	D	Y	W	K	H	M	R	L	E	C	A	I	Y	Y	
E2-2	34–48	K	H	M	R	L	E	C	A	I	Y	Y	K	A	R	E	
	40–54	C	A	I	Y	Y	K	A	R	E	M	G	F	K	H	I	
	47–61	R	E	M	G	F	K	H	I	N	H	Q	V	V	P	T	
	52–66	K	H	I	N	H	Q	V	V	P	T	L	A	V	S	K	
	56–70	H	Q	V	V	P	T	L	A	V	S	K	N	K	A	L	
E2-3	60–74	P	T	L	A	V	S	K	N	K	A	L	Q	A	I	E	
	68–82	K	A	L	Q	A	I	E	L	Q	L	T	L	E	T	I	
	73–88	I	E	L	Q	L	T	L	E	T	I	Y	N	S	Q	Y	S
	80–94	E	T	I	Y	N	S	Q	Y	S	N	E	K	W	T	L	
	85–99	S	Q	Y	S	N	E	K	W	T	L	Q	D	V	S	L	
E2-4	90–104	E	K	W	T	L	Q	D	V	S	L	E	V	Y	L	T	
	94–108	L	Q	D	V	S	L	E	V	Y	L	T	A	P	T	G	
	99–114	L	E	V	Y	L	T	A	P	T	G	C	I	K	K	H	G
	108–122	G	C	I	K	K	H	G	Y	T	V	E	V	Q	F	D	
	113–128	H	G	Y	T	V	E	V	Q	F	D	G	D	I	C	N	T
E2-5	119–134	V	Q	F	D	G	D	I	C	N	T	M	H	Y	T	N	W
	127–141	N	T	M	H	Y	T	N	W	T	H	I	Y	I	C	E	
	132–146	T	N	W	T	H	I	Y	I	C	E	E	A	S	V	T	
	136–150	H	I	Y	I	C	E	E	A	S	V	T	V	V	E	G	
	143–157	A	S	V	T	V	V	E	G	Q	V	D	Y	Y	G	L	
E2-6	150–164	G	Q	V	D	Y	Y	G	L	Y	Y	V	H	E	G	I	
	155–170	Y	G	L	Y	Y	V	H	E	G	I	R	T	Y	F	V	Q
	162–177	E	G	I	R	T	Y	F	V	Q	F	K	D	D	A	E	K
	166–180	T	Y	F	V	Q	F	K	D	D	A	E	K	Y	S	K	
	176–190	E	K	Y	S	K	N	K	V	W	E	V	H	A	G	G	
E2-7	181–196	N	K	V	W	E	V	H	A	G	G	Q	V	I	L	C	P
	190–204	G	Q	V	I	L	C	P	T	S	V	F	S	S	N	E	
	197–211	T	S	V	F	S	S	N	E	V	S	S	P	E	I	I	
	203–217	N	E	V	S	S	P	E	I	I	R	Q	H	L	A	N	
	208–222	P	E	I	I	R	Q	H	L	A	N	H	P	A	A	T	
E2-8	212–226	R	Q	H	L	A	N	H	P	A	A	T	H	T	K	A	
	225–239	K	A	V	A	L	G	T	E	E	T	Q	T	T	I	Q	
	236–250	T	T	I	Q	R	P	R	S	E	P	D	T	G	N	P	
	245–260	P	D	T	G	N	P	C	H	T	T	K	L	L	H	R	D
	254–268	T	K	L	L	H	R	D	S	V	D	S	A	P	I	L	
E2-9	260–274	D	S	V	D	S	A	P	I	L	T	A	F	N	S	S	
	265–280	A	P	I	L	T	A	F	N	S	S	H	K	G	R	I	N
	277–291	G	R	I	N	C	N	S	N	T	T	P	I	V	H	L	
	286–301	T	P	I	V	H	L	K	G	D	A	N	T	L	K	C	L
	296–310	N	T	L	K	C	L	R	Y	R	F	K	K	H	C	T	
E2-10	301–315	L	R	Y	R	F	K	K	H	C	T	L	Y	T	A	V	
	309–323	C	T	L	Y	T	A	V	S	S	T	W	H	W	T	G	
	317–331	S	T	W	H	W	T	G	H	N	V	K	H	K	S	A	
	324–338	H	N	V	K	H	K	S	A	I	V	T	L	T	Y	D	
E2-11	330–344	S	A	I	V	T	L	T	Y	D	S	E	W	Q	R	D	
	335–350	L	T	Y	D	S	E	W	Q	R	D	Q	F	L	S	Q	V
	344–359	D	Q	F	L	S	Q	V	K	I	P	K	T	I	T	V	S
	350–365	V	K	I	P	K	T	I	T	V	S	T	G	F	M	S	I

### T-cell Proliferation Assay

Proliferation assays were performed in quadruplicate with PBMCs (2×10^5^/per well) as previously described [33] and the median value of cpm was calculated. SI were calculated as counts per minute (cpm) in peptide-stimulated cells/cpm in negative control wells (without peptide). Proliferative responses with SI >3 were scored as positive, provided that cpm in the negative control was above 500. Negative controls were PBMC cultured in RPMI 1640–10% heat-inactivated human AB serum. Positive controls were PBMCs activated by phorbol myristate acetate and ionomycine (Sigma).

### Intracellular Cytokine Staining

Cells were incubated overnight at 37°C with 5 µM peptides in RPMI 1640+10% heat-inactivated human AB serum. Brefeldin A (Sigma, at 10 µg/ml final concentration) was added for 4 h at 37°C. Cells were washed in PBS 5% FCS, stained with CD4 Peridinin Chlorophyll Protein (PERCP, Becton Dickinson BD, clone SK3), lysed and permeabilized in buffer (BD) for 10 minutes. After washes, staining was performed for 30 minutes at room temperature in the dark using anti-IFNγ-FITC (BD, clone 25723.11) and anti-IL2-PE antibodies (BD, clone 5344.111). Analyses were done with a FACSCalibur (BD Bioscience). Flow cytometry data were analyzed with CellQuest Pro. For each cytokine or cytokine combinations, results were expressed as percent of cytokine-expressing cells in CD4 positive T-cell subset and compared to negative controls. Positive control antigens were a mixture of short and large influenza, Epstein-bar virus and cytomegalovirus peptides (NeoMPS). Responses were considered positive when the percentages of CD4+ T-cells synthesizing IFNγ and/or IL2 increased by at least 0.2% in the presence of the peptide as compared to negative control. The combination of the two cytokines allows quantifying single producer (IFNγ or IL2 only) and dual producer (IFNγ and IL-2) CD4+ T-cells [26].

### *Ex vivo* ELISPOT Assay for Single Cell IFNγ Release

IFNγ ELISPOT assays were performed as previously described [33]. Negative controls were PBMC incubated in complete medium alone. Positive controls were phorbol myristate acetate- and ionomycine-stimulated cells. Only large spots with fuzzy borders were scored as IFNγ-SFC. Responses were considered significant when the mean number of SFC by 10^6^ cells in the 3 experimental wells was >3-fold the mean number of SFC in the negative control wells (PBMC alone) and >30 SFC/10^6^ cells.

### Statistical Analysis

Fisher exact test was used to evaluate whether HPV-specific T-cell proliferations are statistically different between the groups of women with or without HPV-related symptoms.
